# More Enemy War-Cries: Lay Control

**Published:** 1918-01-19

**Authors:** 


### NURSING AFFAIRS IN IRELAND.

### More Enemy War-Cries: Lay Control.

No morsel is so sweet to the propagandist of faction as the expression, "lay control." When all other arguments are exhausted the orator puts on the black cap, and in pronouncing these words pronounces sentence. Lay control is the capital offence.

The term "lay control" was coined in the mint of the enemy, and is intended, not to appeal to the intellect, not to convince, but to create a false impression, to arouse suspicion, and correspond exactly with a wink or a nod of disparagement about somebody whose character might be spotless as the snow. All these enemy war-cries bear this common characteristic, that they are slanderous suggestions, winks, nods, innuendoes, without any attempt at proof or demonstration. Let us examine and analyse this particular war-cry. The College of Nursing consists of trained-nurse members and a Council. Under this Council are local Boards, one for Scotland and one for Ireland. The proportion of laymen serving on Council or Boards is altogether negligible so far as voting powers are concerned. The Irish Board, for example, consists of twenty-eight members, two of whom are laymen, the remaining twenty-six being doctors and nurses. As all the members of the Council and the Scottish and Irish Boards will ultimately be elective, the lay element may be extinguished if the trained nurses who are the members so desire. But the fundamental fact remains that, lay or professional, neither the Council nor the Boards, collectively or individually, have the power, if they had the desire, to exercise the smallest "control" over the members.

Here are two recent typical references to lay control by prominent faction propagandists, and it will be seen that in neither instance are the facts discussed, but, instead, suggestions are made with the intention of deceiving and of creating a false impression :—

Miss Brodrick : "The butterflies of the aristocracy, the gold-bugs of the plutocracy, have had their day . . Money and social position could accomplish most things, and the cloven hoof was evident in the College of Nursing."

Mrs. Fenwick : "So long as they would eat out of the hand of their employers and accept charity from the mercantile aristocracy they would remain in a most undignified position."

What these references to "employers" are intended to convey is, I presume, that the College Council are the employers of nurses, which, of course, is untrue. The gold-bug, likewise, is intended to call up the image of an Oriental despot with his millions of slaves. It is worthy of note that not one of the demagogues has ever attempted to set forth by what means the layman or the professional people who are members of the Council of the College of Nursing can, by virtue of their position, hope to benefit themselves, or to benefit anybody, except the nurses. Nor has it been mentioned by what malevolent desire they can have been inspired in permitting themselves to be enrolled as members of the Council. As Miss Brodrick probably knows, the first thing that a policeman seeks when a misdemeanour is brought under his notice is a probable motive. What is the motive of the Council of the College? Now, in Ireland there is not a single hospital Board which does not consist partly, at least, of philanthropic laymen. Probably the same holds in Great Britain. In these cases the term "lay control" is used with perfect accuracy, and so is the word "employer." The hospital Boards prescribe the wages, the working hours, the holidays, and all the other conditions of service of hospital nurses. They control and employ. But let there be no confusing of the issues. Let there be no possibility of the enemy persuading the honest nurse that the College is an actual or potential tyrant, any more than Germany is able to persuade the nations of the world that Britain is the tyrant of the seas.

